# Tobacco control policies and respiratory conditions among children presenting in primary care

**DOI:** 10.1038/s41533-024-00369-8

**Published:** 2024-05-16

**Authors:** Timor Faber, Luc E. Coffeng, Aziz Sheikh, Irwin K. Reiss, Johan P. Mackenbach, Jasper V. Been

**Affiliations:** 1 Division of Neonatology, Department of Neonatal and Paediatric Intensive Care, Erasmus MC Sophia Children’s Hospital, University Medical Centre Rotterdam, Rotterdam, The Netherlands; 2 Department of Public Health, Erasmus MC, University Medical Centre Rotterdam, Rotterdam, The Netherlands; 3 Asthma UK Centre for Applied Research Centre of Medical Informatics, Usher Institute, The University of Edinburgh, Edinburgh, UK; 4 Health Data Research UK BREATHE Hub, Edinburgh, UK; 5 Department of Obstetrics and Gynaecology, Erasmus MC Sophia Children’s Hospital, University Medical Centre Rotterdam, Rotterdam, The Netherlands

**Keywords:** Asthma, Health policy, Epidemiology, Epidemiology, Paediatric research

## Abstract

Tobacco control policies can protect child health. We hypothesised that the parallel introduction in 2008 of smoke-free restaurants and bars in the Netherlands, a tobacco tax increase and mass media campaign, would be associated with decreases in childhood wheezing/asthma, respiratory tract infections (RTIs), and otitis media with effusion (OME) presenting in primary care. We conducted an interrupted time series study using electronic medical records from the Dutch Integrated Primary Care Information database (2000–2016). We estimated step and slope changes in the incidence of each outcome with negative binomial regression analyses, adjusting for underlying time-trends, seasonality, age, sex, electronic medical record system, urbanisation, and social deprivation. Analysing 1,295,124 person-years among children aged 0–12 years, we found positive step changes immediately after the policies (incidence rate ratio (IRR): 1.07, 95% CI: 1.01–1.14 for wheezing/asthma; IRR: 1.16, 95% CI: 1.13–1.19 for RTIs; and IRR: 1.24, 95% CI: 1.14–1.36 for OME). These were followed by slope decreases for wheezing/asthma (IRR: 0.95/year, 95% CI: 0.93–0.97) and RTIs (IRR: 0.97/year, 95% CI: 0.96–0.98), but a slope increase in OME (IRR: 1.05/year, 95% CI: 1.01–1.09). We found no clear evidence of benefit of changes in tobacco control policies in the Netherlands for the outcomes of interest. Our findings need to be interpreted with caution due to substantial uncertainty in the pre-legislation outcome trends.

## Introduction

Tobacco smoking remains the world’s most important preventable cause of morbidity and premature mortality^1^. Passive exposure to tobacco smoke, or second-hand smoke (SHS) exposure, is responsible for more than one million deaths per year globally, including over 50,000 children under the age of 10^2^. Exposure to SHS also increases the risk of a range of adverse health outcomes in early life, including wheezing/asthma, respiratory tract infections (RTIs), and otitis media with effusion (OME)^3–7^. Substantial evidence now indicates that children benefit from the implementation of comprehensive tobacco control policies—for example, laws that prohibit smoking in indoor public places and workplaces^8,9^. In a recent systematic review and meta-analysis, including data on over 27 million children, we demonstrated that the introduction of smoke-free legislation was associated with a −18% decrease in hospital attendance for lower RTIs (95% confidence interval (CI) −33 to −4), and a −10% decrease in hospital attendance for asthma (95% CI −17 to −3) among children^8^. Effects on childhood OME have been assessed in one study, but no significant changes were found^10^.

Most of this research has focused on severe childhood health outcomes such as hospitalisations, which reflect only part of the burden of disease caused by tobacco smoke exposure. Children with milder respiratory symptoms are more likely to be diagnosed in primary care. There is inconclusive evidence on whether respiratory health outcomes in primary care are directly affected by tobacco control policies. We are aware of one study that aimed to determine the effects of a comprehensive smoke-free law on adverse paediatric respiratory outcomes in primary care in the UK^11^. This found no significant changes in wheezing/asthma and RTI diagnoses in children in primary care after the introduction of national comprehensive smoke-free legislation, whereas other studies did find substantial reductions in hospitalisations for these outcomes in England and Scotland^12–14^. From this study, it is unclear whether outcomes in primary care are inherently less affected or not affected by smoke-free laws than outcomes in hospital care, or whether these changes have not been detected due to methodological reasons^11^.

In the Netherlands, about 7% of all children aged 0–11 were exposed to tobacco smoke in some way in 2020^15^. Although the smoking prevalence among adults has decreased during the last decade, 20% of the adult Dutch population reported to smoke daily or occasionally in 2020, with the highest percentages among people of childbearing age (aged 18–49)^15^. Following on from regulation introduced in 2004 which prohibited smoking in most enclosed workplaces and public places, the Dutch government introduced legislation in 2008 mandating smoke-free restaurants and bars. This introduction was accompanied by a tobacco tax increase and a mass media campaign. Given the overwhelming evidence linking smoke-free legislation to reduced hospitalisations for respiratory disorders among children, we were interested in whether tobacco control policies also directly affect the actual incidence of these outcomes in primary care. In this study, we examined the hypothesis that the introduction of these tobacco control policies was associated with reductions in the incidence of general practitioner (GP) diagnoses of wheezing/asthma, RTIs, and OME in children aged ≤12 years.

## Results

### Patient population

**Fig. 1:**
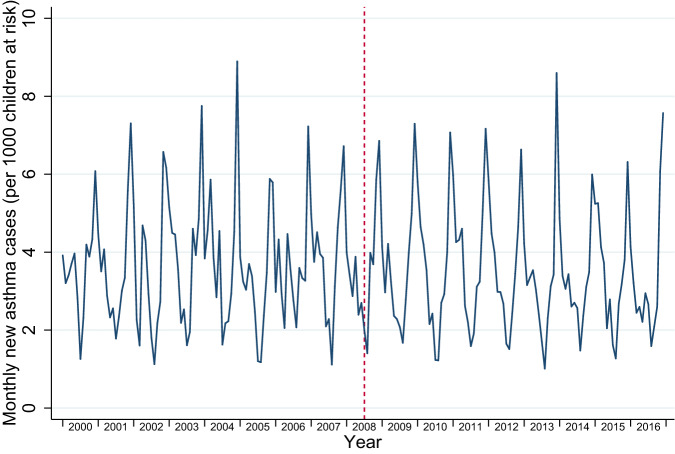
Incidence trends in new wheezing/asthma diagnoses.

**Fig. 2:**
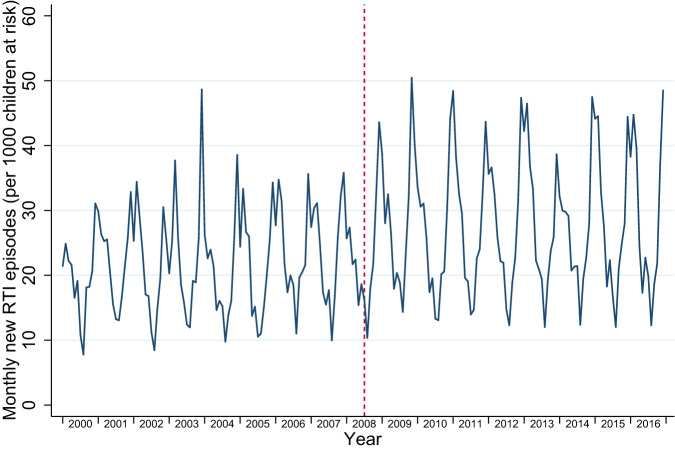
Incidence trends in new respiratory tract infection (RTI) diagnoses.

**Fig. 3:**
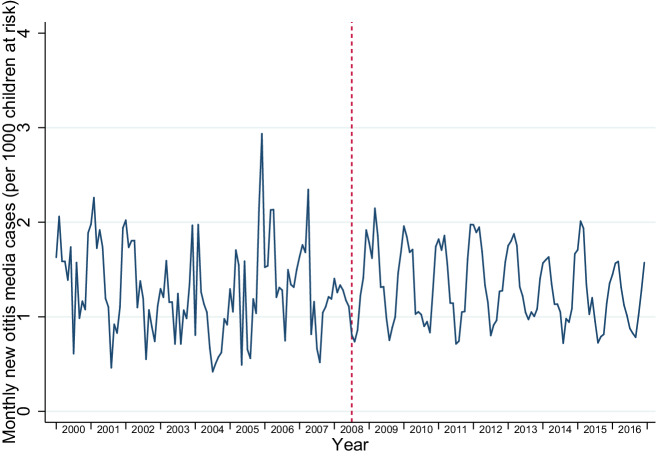
Incidence trends in new otitis media with effusion diagnoses.

Between 2000 and 2016, there were 917,532 person-years at risk for wheezing/asthma, 1,295,124 person-years at risk for RTIs, and 1,245,885 person-years at risk for OME. During these at-risk periods there were 38,430 new diagnoses of wheezing/asthma, 417,597 new diagnoses of RTIs, and 19,266 new diagnoses of OME. When separately examining URTIs and LRTIs, including LRTIs that directly followed an URTI, there were 377,418 URTIs and 48,501 LRTIs. The distribution of person-years at risk and, consequently, the number of new diagnoses for all of our outcomes were heavily skewed towards the post-intervention period: most GP practices joined IPCI after the policies were introduced, resulting in roughly 10 times more person-years included in the post-intervention period (Supplementary Table 1 and Supplementary Fig. 1). Younger children (aged 0–4 years) had higher mean incidence rates of all outcomes (Table 1; Supplementary Table 2). Figures 1–3 display the monthly rates of new wheezing/asthma, RTI, and OME diagnoses over the study period. The seasonal variations of these outcomes within each year appeared to be consistent over the entire study period.

**Table 1:** Event counts and person-years at risk by sex, age group, social deprivation, and urbanisation.

	Outcomes								
	Wheezing/asthma			Respiratory tract infections			Otitis media with effusion		
	Events	Person-years at risk	Crude incidence per 1000 person-months	Events	Person-years at risk	Crude incidence per 1000 person-months	Events	Person-years at risk	Crude incidence per 1000 person-months
Total	38,430	917,532	3.49	417,597	1,295,124	26.87	19,266	1,245,885	1.29
*Sex*									
Female	16,522	472,865	2.91	198,333	632,843	26.12	9226	609,519	1.26
Male	21,908	444,667	4.11	219,264	662,281	27.59	10,040	636,366	1.31
*Age group*									
0–4 years	30,312	363,094	6.96	281,191	475,259	49.30	10,245	460,563	1.85
5–12 years	8118	554,438	1.22	136,406	819,865	13.86	9021	785,322	0.96
*Social deprivation*									
Yes	2107	41,232	4.26	26,842	60,379	37.05	632	58,569	0.90
No	31,371	754,268	3.47	345,005	1,070,955	26.85	15,890	1,030,203	1.29
Missing	4952	122,031	3.38	45,750	163,790	23.28	2744	157,113	1.46
*Urbanisation*									
Urban	13,015	295,330	3.67	142,481	413,179	28.74	5770	397,887	1.21
Rural	11,178	302,167	3.08	132,217	422,515	26.08	6507	406,891	1.33
Missing	14,237	320,035	3.71	142,899	459,430	25.92	6989	441,107	1.32

### Associations between introduction of policy and outcomes

The 2008 policy changes were associated with an immediate step increase in new wheezing/asthma diagnoses (incidence rate ratio (IRR) 1.07; 95% confidence interval (CI) 1.01–1.14), followed by a slope decrease (IRR 0.95 per year; 95% CI 0.93–0.97) (Table 2). Similarly, for RTI diagnoses there was a temporary increase (IRR 1.16; 95% CI 1.13–1.19), followed by a slope decrease (IRR 0.97 per year; 95% CI 0.96–0.98). For OME incidence, the policy change was associated with both a step increase (IRR 1.24; 95% CI 1.14–1.36), and a slope increase (IRR 1.05 per year; 95% CI 1.01–1.09) (Table 2).

**Table 2:** Multivariable negative binomial regression analyses for wheezing/asthma, RTIs, and OME.

	Wheezing/asthma		RTIs		URTIs		LRTIs		OME	
	IRR	95% CI	IRR	95% CI	IRR	95% CI	IRR	95% CI	IRR	95% CI
Pre-2008 trend	1.02	1.00–1.04	1.03	1.02–1.04	1.03	1.02–1.04	1.08	1.05–1.10	0.92	0.88–0.95
Step change	1.07	1.00–1.14	1.16	1.13–1.19	1.17	1.14–1.20	1.12	1.05–1.19	1.24	1.14–1.36
Slope change	0.95	0.93–0.97	0.97	0.96–0.98	0.98	0.96–0.99	0.87	0.85–0.89	1.05	1.01–1.09

Analyses were adjusted for the underlying time-trend, seasonality, age group, sex, EMR software system, urbanisation level, and social deprivation.

Both URTIs and LRTIs showed a step increase following the 2008 policy change (URTIs: IRR 1.17; 95% CI 1.14–1.20; LRTIs: IRR 1.12; 95% CI 1.05–1.19), again followed by a slope decrease (URTIs: IRR 0.98 per year; 95% CI 0.96–0.99; LRTIs: IRR 0.87 per year; 95% CI 0.85–0.89) (Table 2).

The sensitivity analyses of (1) complete cases only, (2) without urbanisation level and social deprivation, and (3) the data with imputed values for missing entries of urbanisation level and social deprivation provided results similar to the primary analyses (Supplementary Tables 3–5).

Using a counterfactual scenario, we estimated that in the first eight years following the policy change 0.68 new wheezing/asthma diagnoses (95% CI 0.65–0.71) and 0.80 new RTI diagnoses per 1000 person-months were averted (95%CI 0.64-0.97). In contrast, following the policy implementation an additional 0.47 new OME diagnoses per 1000 person-months were made during this period (95% CI 0.46–0.47).

## Discussion

A policy change involving implementation of smoke-free hospitality businesses, a mass media campaign, and a tobacco tax increase in the Netherlands was followed by a temporary increase and a subsequent gradual decrease in asthma diagnoses and RTIs in children. This translated into a net reduction in the number of asthma diagnoses and RTIs over the post-implementation period. Unexpectedly, OME diagnoses among children increased following the policy. Our findings need to be interpreted with caution due to the limited amount of data in the pre-legislation period, introducing substantial uncertainty in the pre-legislation trends.

Exposure to tobacco smoke undisputedly leads to higher risk of adverse respiratory outcomes in both children and adults, including RTIs and asthma^1,16^. There is also substantial evidence that the implementation of comprehensive smoke-free legislation has a positive impact on hospitalisations for RTIs and asthma in both children and adults, with the more comprehensive laws being more effective^8,17^. The most likely pathways for these effects are reduced SHS exposure in public and in the home environment as a result of behavioural changes in society^18^.

Although there was a net reduction in the number of asthma and RTI cases following the policy under study, the initial temporary increases in these diagnoses as well as the sustained increase in OME diagnoses are counterintuitive against this background. A number of factors could help explain why this is the case. First, the Dutch tobacco control approach might not have been immediately effective due to its lack of comprehensiveness and its phased approach, as well as lack of compliance^19^. For example, whereas the WHO urges for restaurants and bars to be completely smoke-free, the Dutch law allowed for indoor smoking rooms, which have been found to be ineffective in protecting from tobacco smoke exposure^20,21^. Also, one year after the implementation of the smoke-free restaurants and bars law, 36% of smokers still noticed smoking in bars outside the designated smoking areas in the Netherlands, compared to 1.7% in England and 5.4% in Ireland, for example, where the national smoke-free policy included all public places with no exceptions^22^. Compared to 11 other countries included in the International Tobacco Control Policy Evaluation Project, Dutch people who smoked were at the time also the least knowledgeable or concerned about the harms of tobacco smoking and SHS exposure (in 2010, only 61% of them agreed that cigarette smoke is dangerous to non-smokers compared to 96% of people who smoked in France, 89% in Germany, and 83% in the UK)^23^. In a previous study assessing perinatal health outcomes in the Netherlands, there was a relative lack of impact of the Dutch smoke-free law as compared to England, where the law had been much more comprehensive and compliance was excellent^24,25^. Despite this, exposure of young children to tobacco smoke in the home, likely the primary source of exposure, decreased substantially across the study period^26,27^. In the absence of more detailed trends in tobacco smoke exposure in various places at the time, we lack ability to assess causality of the observed temporal association between the policy change and short-term increases in these respiratory diagnoses.

Previous research suggests that tobacco smoke exposure has a larger impact on RTI severity and asthma exacerbations than on RTI and asthma incidence per se^10,28,29^. In line with this, studies in England have demonstrated a positive impact of the smoke-free law on paediatric hospital admissions, but not on GP diagnoses for asthma and RTIs^11,12,14^. In our study, we were limited to the EMR diagnosis and medication codes registered by the GPs, and therefore had limited ability to distinguish between asthma/RTI diagnosis, treatment, or exacerbation. Similarly, we were unable to analyse Dutch national hospital admissions data due to temporal data limitations of the respective database^30^.

Lastly, other changes over time might have influenced our outcomes of interest that we could not correct for in our analyses. In 2006, the Dutch government introduced a fundamental reform of the health insurance system. Among many changes, this reform led to a change in the remuneration system for GPs^31^, a gradual increase in the supply and use of EMR systems, and the systematic use of ICPC disease codes and ATC medication codes for reimbursement. Although it is possible or even likely that coding and registering behaviour changed during our study period, we were unable to evaluate whether and how this may have affected our results. In addition, there might have been other changes affecting the incidence of our outcomes of interest over time that we are not aware of. Previous work in this field has demonstrated that lack of accounting for unanticipated breakpoints in time series may produce potentially spurious findings^32^. Due to the inherent restrictions of our time series as a result of the low number of GP practices contributing data pre-legislation, as outlined earlier, we did not undertake breakpoint detection in the current study.

To the best of our knowledge, this is the first study aimed at determining the association between introduction of tobacco control policies and child health outcomes in primary care in the Netherlands. We analysed over one million person-years of data while adjusting for important individual-level confounders. At the same time, it is important to note that our study has significant constraints. With the intervention being a national policy implementation, we lacked a true control group and were unable to randomise treatment allocation. Even though we have used optimal methods to evaluate this public health intervention, our study design is still quasi-experimental, and therefore inherently subject to bias^33^. In our attempt to capture the true association between the policy change and our selected outcomes, we adjusted our analyses for potential confounding factors that could have influenced our results. However, our data were particularly unbalanced in relation to the timing of the intervention, with very few GPs and participants contributing data to the pre-legislation period versus the post-legislation period (93,104 versus 824,428 person-years at risk for wheezing/asthma, 123,702 versus 1,171,421 person-years at risk for RTIs, and 120,719 versus 1,125,167 person-years at risk for OME). This complicated proper estimation of the pre-legislation trends and hence limited the ability of our models to reliably determine the intervention effects. In addition to the uncertainty in the parameter estimates caused by the low pre-post data ratio, our results may be biased by temporal changes in registration behaviour by GPs, as outlined earlier. This may have resulted in an underestimation of number of respiratory diseases in children in the pre-intervention period, possibly overestimating the step changes as a result.

In conclusion, there was a modest net reduction in paediatric asthma and RTI diagnoses following a national set of tobacco control policies in the Netherlands. This was however preceded by an unexpected temporary increase in diagnoses and accompanied by a sustained increase in OME diagnoses. Although the exact mechanisms underlying these observations remain unclear, limitations of the underlying data may have biased our findings.

## Methods

### Study design

We conducted interrupted time series (ITS) analyses to determine the associations between the implementation of the July 2008 Dutch national smoke-free restaurants and bars policy^34^, and changes in the incidence rates of wheezing/asthma, RTIs, and OME among children.

### Setting

We used data from the Integrated Primary Care Information (IPCI) database, a longitudinal database of electronic medical records (EMRs) from primary care practices in the Netherlands. The IPCI database was founded in 1989 and is acclaimed for its comprehensive medical information^35^. The data in IPCI are an open cohort: GP practices and patients may enter and leave the database at any time. As the EMRs were anonymised, the geographical locations of the patients and GP practices were unknown to the researchers using these data. Details of the IPCI database have been described previously^35^. To perform the ITS analysis around the key year 2008 (when policy changed), we decided a priori to include data from January 2000 through December 2016.

### Participants

For our study, children were included if they were aged ≤12 years and were registered with a GP practice in the IPCI database for at least six consecutive months during the study period. Children who entered the GP practice as a newborn (i.e., within 6 months after birth) and who contributed at least 6 months of data were eligible. Participants were followed-up until they turned 13 years of age, changed to a GP practice not registered in IPCI, left the country, or died. The age cut-off of 12 years of age was selected to minimise the potential confounding effect of active smoking among participants.

### Outcomes

Our primary outcomes of interest were the monthly incidence of new diagnoses of wheezing/asthma, RTIs, and OME. Our secondary outcomes of interest were the monthly incidence of new diagnoses of upper RTIs (URTI) and lower RTIs (LRTI).

We specified a new diagnosis of wheezing/asthma when a relevant diagnostic International Classification of Primary Care (ICPC) code was recorded in a child’s medical records and/or when a prescription for asthma-related medication was recorded in the medical records of a child who had no previous recording of wheezing/asthma diagnostic codes or asthma-related prescriptions (the relevant ICPC codes are listed in the Online Supplement). Children did not have a new diagnosis if wheezing/asthma was recorded before or on the first day of registration with an IPCI practice, as they were considered to be prevalent cases of wheezing/asthma. Asthma-related medications included selective beta-2 adrenoreceptor agonists, anticholinergics, inhalation corticosteroids, and leukotriene receptor antagonists (for details, see the Online Supplement).

Incident diagnoses of RTIs were specified when an ICPC code for either an URTI or LRTI was recorded in a child’s medical records. As most RTIs among children resolve within 15 days, we considered a new RTI diagnosis only when registered at least 21 days after any prior RTI consultation, to minimise repeated registration of GP visits for the same RTI episode^36^.

We defined children to have a new OME diagnosis if the relevant ICPC code was recorded in a child’s medical records and there was no prior registration of an OME code in the preceding six months. We applied this six-month window to exclude repeated GP visits for the same episode of OME as around 72% of OME cases (95% CI 68–76) in children are known to resolve spontaneously within 6 months^37^.

We considered separate diagnoses of URTIs and LRTIs as secondary outcomes. Similar to RTIs in general, we defined multiple visits within 14 days as belonging to the same URTI/LRTI diagnosis. However, if an URTI was followed by a LRTI within 14 days, both the URTI and LRTI were recorded as separate diagnoses.

The GPs used codes from the first edition of the ICPC to register disease diagnoses in EMRs^38^. Medication codes were registered using the World Health Organization’s (WHO) Anatomical Therapeutic Chemical (ATC) codes^39^.

### Person-time at risk

A child was considered to be at risk for an outcome in a particular month if:

- The child had no prior registration of an asthma code, including in registration of prior disease history in patients newly entering GP practice (Online Supplement) [for wheezing/asthma]
- The child had no prior issuing of wheezing/asthma-related medication prescription, including in registration of prior medication history in patients newly entering GP practice (Online Supplement) [for wheezing/asthma]
- No OME code was registered within the six preceding months (Online Supplement) [for OME].

### Main predictor of interest

The intervention was the introduction of a law on 1 July 2008 prohibiting smoking in hospitality venues, i.e., hotels, bars and restaurants, sports, arts and culture venues, amusement arcades, tobacco shops, and international passenger transport^34^. This law allowed for designated indoor smoking areas^34^. The law was accompanied by a mass-media campaign and an 8% excise tax increase on tobacco products^40^.

### Confounding factors

In our analyses, we adjusted for the following potential confounders: the underlying time-trend in the outcome, seasonality (i.e. month of diagnosis; categorical), age group (0–4; 5-12 years of age), sex (female; male), EMR software system (HetHIS; Medicom; MicroHIS, MicroHIS Old; Mira; Promedico ASP; Promedico VDF Old; WebHIS Zorgdossier), urbanisation level (urban: ≥1500 inhabitants per km^2^; rural: <1500 inhabitants per km^2^), and social deprivation (yes: living in an area in the bottom 5% of that year’s national list of postal codes ranked according to social deprivation; no: in the top 95% of this list).

Due to a high proportion of missing values for urbanisation and social deprivation (i.e. 35% and 13%, respectively), we re-coded missing values into a third category, i.e. urban, rural, missing for urbanisation level, and yes, no, missing for social deprivation. Urbanisation level and social deprivation were completely missing for the years 2000–2003, as registration of postal code only started in 2004.

### Statistical methods

Our primary analysis was an ITS negative binomial regression analysis in which we investigated the association between the implementation of the tobacco control policies in 2008 and the change in incidence of each of our outcomes of interest. A negative binomial model (rather than a Poisson model) was adopted to account for over-dispersion (variance > mean) of case incidences over time.

Our models allowed for both an immediate change in level of the outcomes (step change) using a dichotomous time-variant dummy variable, as well as a gradual change in temporal trend of our outcomes (slope change) using an interaction term between the dummy variable and year (continuous). We accounted for seasonality (categorical variable for month) and the underlying temporal incidence trend (year as a continuous variable, centred at 1 July 2008). We modelled the underlying temporal trend via linear, quadratic, and cubic B-splines in separate models to account for possible non-linearity. We selected the optimal model using Aikaike’s and Bayesian Information Criteria (AIC and BIC, respectively). Using comparison of predicted values from our models and counterfactual models with step and slope changes set to zero, we estimated the absolute number of events averted across the post-legislation study period for each outcome. We performed all analyses using Stata SE 15.1 (Statacorp, TX).

### Sensitivity analyses

In post hoc sensitivity analyses, we assessed the potential impact of the substantial proportion of children with missing postal code data on our findings via: 1. including only cases with complete data on all covariates, 2. not adjusting for urbanisation level and social deprivation, and 3. imputing values for urbanisation level and social deprivation. For the latter purpose we conducted and analysed 20 imputations of these values based on all the available variables in the dataset, using the Stata commands *mi impute monotone (logit)* and *mi estimate*.

### Ethical considerations and reporting

The IPCI Governance Board approved this study (no. 03/2015). The IPCI data are not subject to the Medical Research Involving Human Subjects Act (WMO) and therefore do not require approval from a medical research ethics committee. We conducted this study using a pre-specified study protocol. We performed all methods in accordance with the relevant guidelines and regulations. Meta-data and data are property of IPCI. Researchers who are interested can contact the IPCI project team at www.ipci.nl. Our study protocol and statistical codes are available on request from the corresponding author. We used the STROBE and RECORD guidelines to report our findings.

### Reporting summary

Further information on research design is available in the Nature Research Reporting Summary linked to this article.

### Supplementary information

Online Supplement

Reporting Summary

## Data Availability

The data belong to IPCI and are only available through them. The code syntaxes from the current analyses are available from the corresponding author on reasonable request.
