# Supplementary material for: Tobacco control policies and respiratory conditions among children presenting in primary care
Source: NPJ Prim Care Respir Med. 2024 May 16;34:11. doi: 10.1038/s41533-024-00369-8 (PMC11099007; doi:10.1038/s41533-024-00369-8)
Supplement: Supplementary file 1 — Online Supplement [file 41533_2024_369_MOESM1_ESM.pdf]

SUPPLEMENTARY INFORMATION

Tobacco control policies and respiratory conditions among children presenting in primary care

Timor Faber, Luc E Coffeng, Aziz Sheikh, Irwin K Reiss, Johan P Mackenbach, and Jasper V Been

Index

List of ICPC codes for wheezing/asthma 3

List of ATC codes for asthma medication 4

List of ICPC codes for respiratory tract infections 5

List of ICPC codes for otitis media with effusion 7

Supplementary Table 1: Event counts and patient-years observed by pre- and post-intervention period 8

Supplementary Figure 1: Person-months at risk over time 9

Supplementary Table 2: Upper and lower RTI event counts and patient-years observed by sex, age group, social deprivation, and urbanisation 10

Supplementary Table 3 - Multivariable negative binomial regression analyses for wheezing/asthma, RTIs, and otitis media with effusion with complete cases of social deprivation and living environment 11

Supplementary Table 4 - Multivariable negative binomial regression analyses for wheezing/asthma, RTIs, and otitis media with effusion without social deprivation and living environment as covariates12

Supplementary Table 5 - Multivariable negative binomial regression analyses for wheezing/asthma, RTIs, and otitis media with effusion with imputed values for social deprivation and living environment 13

List of ICPC codes for wheezing/asthma

ICPC-1-2013 codes

R96 – Asthma

- R96.00 - Asthma
- R96.01 – Hyperactivity airways
- R96.02 – Allergic asthma
- R96.03 – Exercise-induced asthma
- R96.04 – Asthma attack
- R96.05 – Status asthmaticus
- R96.06 – Other/non-specified asthma

R03.00 – Wheezing

List of ATC codes for asthma medication

Bronchodilators:

Long- and short-acting beta-2-adrenoreceptor agonists

R03AC – Selective beta-2-adrenoreceptor agonists (HT)

R03AC02	salbutamol
R03AC03	terbutaline
R03AC04	fenoterol
R03AC12	salmeterol
R03AC13	formoterol

Anticholinergics

R03BB – Anticholinergics (HT)

R03BB01	ipratropium bromide
R03BB04	tiotropium bromide

Inhalation corticosteroids:

R03BA – Glucocorticoids (HT)

R03BA01	beclometasone
R03BA02	budesonide
R03BA05	fluticasone
R03BA08	ciclesonide

Drug combinations:

R03AK - Adrenergics in combination with corticosteroids or other drugs, excl. anticholinergics (HT)

R03AK06	salmeterol and fluticasone
R03AK03	fenoterol and other drugs for obstructive airway diseases
R03AK04	salbutamol and other drugs for obstructive airway diseases
R03AK07	formoterol and other drugs for obstructive airway diseases

Leukotriene receptor antagonists:

R03DC - Leukotriene receptor antagonists

R03DC03	montelukast

List of ICPC codes for respiratory tract infections

ICPC-1-2013 codes

Upper OR lower respiratory tract infections
R83.00 – Other infections of respiratory system

In combination with A03.00 – Fever or A02.00 – Chills:
R05.00 – Cough

Acute upper respiratory tract infections
H71.00 – Acute otitis media/myringitis
- H71.01 – Acute otitis media
- H71.02 – Acute myringitis
- H71.03 – Non-specified acute otitis media

H73.00 – Eustachian salpingitis
- H73.01 – Catarrhal inflammation of the Eustachian tube
- H73.02 – Stenosis of the Eustachian tube

R70.00 – Whooping cough
R71.00 – Strep throat
R72.00 – Streptococcal angina/scarlet fever
- R72.01 – Streptococcal angina
- R72.02 – Scarlet fever

R74.00 – Acute upper respiratory infection
- R74.01 – Common cold
- R74.02 – Acute pharyngitis
- R74.03 – Other acute upper respiratory infection

R75.01 – Sinusitis acute
R76.00 – Tonsillitis acute/peritonsillar abscess
- R76.01 – Tonsillitis acute
- R76.02 – Peritonsillar abscess

R77.00 – Acute laryngitis/tracheitis
- R77.01 – Laryngitis subglottica/pseudocroup
- R77.02 – Epiglottitis acute
- R77.03 – Other form of laryngitis/tracheitis

R80.00 – Influenza (proven) without pneumonia

In combination with A03.00 – Fever or A02.00 – Chills:
H01.00 – Ear pain/earache
R01.00 – Pain respiratory system
R07.00 – Sneezing/nasal congestion
R09.00 – Symptom/complaint sinus (including pain)
R21.00 – Symptom/complaint throat

R21.01 – Sore throat
R21.02 – Other symptom/complaint throat
R22.00 – Symptom/complaint tonsils

Acute lower respiratory tract infections
R78.00 – Acute bronchitis/bronchiolitis
R82.00 – Pleurisy/pleural effusion (excluding R70 – Tuberculosis)
R81.00 – Pneumonia
R81.01 – Influenza with pneumonia
R81.02 – Other viral pneumonia
R81.03 – Pneumococcal pneumonia
R81.04 – Pneumonia by mycoplasma pneumoniae
R81.05 – Other bacterial pneumonia
R81.06 – Other/non-specified pneumonia

In combination with A03.00 – Fever or A02.00 – Chills
R25.00 – Abnormal sputum/phlegm
R02.00 – Shortness of breath/dyspnoea

List of ICPC codes for otitis media with effusion

ICPC-1-2013 codes

Otitis media with effusion
H72.00 – Serous otitis media/glue ear

Supplementary Table 1: Event counts and patient-years observed by pre- and post-intervention period

	**Pre-intervention period**	**Post-intervention period**
**Wheezing/asthma**		
Events	4,049	34,381
Person-years observed	93,104	824,428
Mean incidence per 1,000 person-months	3.62	3.48
**RTIs**		
Events	32,652	384,945
Person-years observed	123,702	1,171,412
Mean incidence per 1,000 person-months	22.00	27.38
**OME**		
Events	1,825	17,441
Person-years observed	120,719	1,125,167
Mean incidence per 1,000 person-months	1.26	1.29

Supplementary Figure 1: Person-months at risk over time

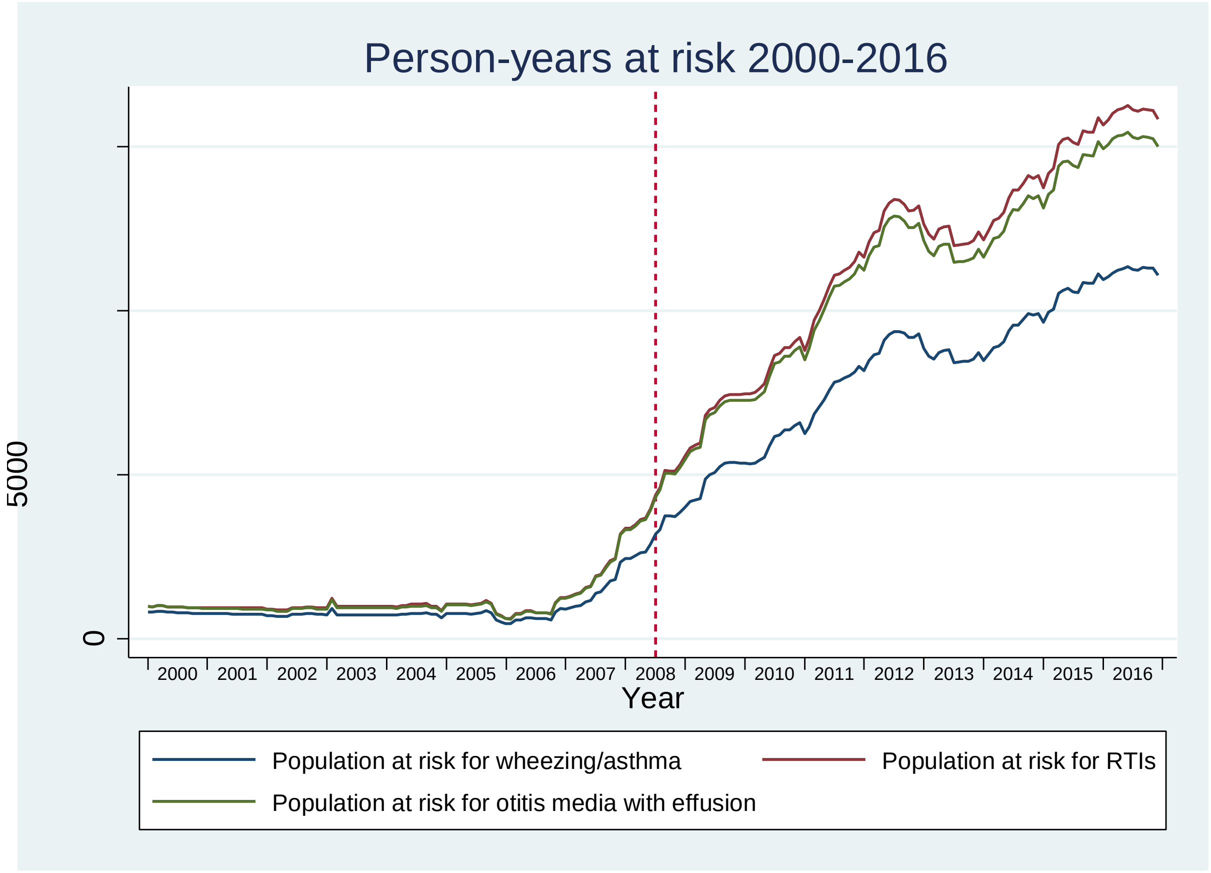

Supplementary Table 2: Upper and lower RTI event counts and patient-years observed by sex, age group, social deprivation, and urbanisation

		Respiratory tract infections		Upper respiratory tract infections		Lower respiratory tract infections	
	**Person-years observed (n)**	**Events (n)**	**Mean incidence per 1,000 person-months**	**Events (n)**	**Mean incidence per 1,000 person-months**	**Events (n)**	**Mean incidence per 1,000 person-months**
**Total**	**1,295,124**	**417,597**	**26.87**	**377,418**	**24.28**	**48,501**	**3.12**
**Sex**							
Female	632,843	198,333	26.12	180,449	23.76	21,514	2.83
Male	662,281	219,264	27.59	196,969	24.78	26,987	3.40
**Age group**							
0–4 years	475,259	281,191	49.30	255,695	44.83	32,638	5.72
5–12 years	819,865	136,406	13.86	121,723	12.37	15,863	1.61
**Social deprivation**							
Yes	60,379	26,842	37.05	24,914	34.39	2,514	3.47
No	1,070,955	345,005	26.85	311,420	24.23	40,676	3.17
Missing	163,790	45,750	23.28	41,084	20.90	5,311	2.70
**Urbanisation**							
Urban	413,179	142,481	28.74	129,803	26.18	15,649	3.16
Rural	422,515	132,217	26.08	119,268	23.52	15,805	3.12
Missing	459,430	142,899	25.92	128,347	23.28	17,047	3.09

Supplementary Table 3 - Multivariable negative binomial regression analyses for wheezing/asthma, RTIs, and otitis media with effusion with complete cases of social deprivation and living environment

	**Step change**			**Slope change**		
	**IRR**	**95% CI**	**P-value**	**IRR**	**95% CI**	**P-value**
**Wheezing/asthma**	1.02	0.94 to 1.12	0.596	0.92	0.86 to 0.97	0.004
**RTIs**	1.19	1.15 to 1.22	<0.001	0.97	0.95 to 0.99	0.007
Upper RTIs	1.19	1.15 to 1.23	<0.001	0.97	0.95 to 0.99	0.017
Lower RTIs	1.23	1.14 to 1.33	<0.001	0.95	0.90 to 1.00	0.071
**Otitis media with effusion**	1.25	1.11 to 1.41	<0.001	1.13	1.05 to 1.22	0.002

Supplementary Table 4 - Multivariable negative binomial regression analyses for wheezing/asthma, RTIs, and otitis media with effusion without social deprivation and living environment as covariates

	**Step change**			**Slope change**		
	**IRR**	**95% CI**	**P-value**	**IRR**	**95% CI**	**P-value**
**Wheezing/asthma**	1.08	1.01 to 1.14	0.022	0.95	0.93 to 0.97	<0.001
**RTIs**	1.16	1.13 to 1.19	<0.001	0.97	0.96 to 0.98	<0.001
Upper RTIs	1.17	1.14 to 1.20	<0.001	0.98	0.97 to 0.99	<0.001
Lower RTIs	1.12	1.05 to 1.19	<0.001	0.87	0.85 to 0.89	<0.001
**Otitis media with effusion**	1.25	1.14 to 1.36	<0.001	1.05	1.01 to 1.09	0.013

Supplementary Table 5 - Multivariable negative binomial regression analyses for wheezing/asthma, RTIs, and otitis media with effusion with imputed values for social deprivation and living environment

	**Step change**			**Slope change**		
	**IRR**	**95% CI**	**P-value**	**IRR**	**95% CI**	**P-value**
**Wheezing/asthma**	1.08	1.01 to 1.15	0.016	0.95	0.93 to 0.97	<0.001
**RTIs**	1.16	1.14 to 1.19	<0.001	0.97	0.96 to 0.98	<0.001
Upper RTIs	1.17	1.14 to 1.20	<0.001	0.98	0.97 to 0.99	<0.001
Lower RTIs	1.12	1.06 to 1.19	<0.001	0.87	0.85 to 0.89	<0.001
**Otitis media with effusion**	1.24	1.14 to 1.35	<0.001	1.05	1.01 to 1.09	0.010
